# The Meaning of Surviving Three Years after a Heart Transplant—A Transition from Uncertainty to Acceptance through Adaptation

**DOI:** 10.3390/ijerph17155434

**Published:** 2020-07-28

**Authors:** Catharina Lindberg, Matilda Almgren, Annette Lennerling, Anna Forsberg

**Affiliations:** 1 Department of Health Sciences, Blekinge Institute of Technology, 374 35 Karlskrona, Sweden; catharina.lindberg@bth.se; 2 Care in High Tech Environments, Institute of Health Sciences at Lund University, 221 00 Lund, Sweden; matilda.almgren@med.lu.se; 3 The Transplant Centre, Sahlgrenska University Hospital, 413 45 Gothenburg, Sweden; annette.lennerling@vgregion.se; 4 Institute of Health and Care Sciences, The Sahlgrenska Academy, University of Gothenburg, 405 30 Gothenburg, Sweden; 5 Department of Thoracic Surgery, Skåne University Hospital, 222 42 Lund, Sweden

**Keywords:** heart transplantation, acceptance, adaptation, phenomenological-hermeneutic, qualitative, transition

## Abstract

The rationale was to longitudinally follow-up interviews performed with heart recipients at their one-year examination in order to deepen the understanding of the meaning of surviving a heart transplant. The aim was to explore the meaning of surviving three years after a heart transplant compared to one year and to identify what constitutes the change process. A phenomenological–hermeneutic method was used. This multicenter study was carried out at the two hospitals in Sweden where heart transplants are performed. A total of 13 heart recipients who survived three years after a heart transplant were invited to participate in this three-year follow-up study and 12 accepted, 3 women and 9 men, with a mean age of 51.25 years. The *naïve* understanding revealed that the heart recipients strongly accepted their life situation and that time had enabled this acceptance of limitations through adaptation. The thematic structural analyses cover six themes illustrating the meaning of acceptance and adaptation, i.e., accepting life as it is, adapting to post-transplant limitations, adapting to a changed body, social adaptation, showing gratitude and trusting oneself and others. In conclusion, achieving acceptance and a solid sense of self-efficacy after heart transplantation is a time-consuming process that involves courage to face and accept the reality and adapt in every life dimension.

## 1. Introduction

Life-threatening heart failure can be successfully treated with heart transplantation (HTx), which is dependent on the availability of donated organs from deceased persons [1]. As the availability of organs is limited, the waiting period can be long and the patient might need mechanical circulatory support (MCS) to survive until HTx. Being an adult MCS-recipient means a liminal existence [2] and vocational adjustment [3]. Young adult patients receiving MCS navigate from helplessness to feeling strong in their new reality [4]. Some patients receiving MSC might prefer to continue it even when transplant becomes an option [5].

Becoming a heart transplant recipient (HTR) is a life changing event that can lead to changes in self-identity [1,6,7]. Of all medical interventions, HTx is without a doubt the one that implies the greatest contrast of emotions, including deep pain, frustration and fear of death together with intense happiness and joy of life [8]. Previous qualitative research on HTRs has taken the form of occasional interviews [9,10,11,12]. There is thus a need for a prospective, qualitative design to grasp the presumed transition, as in this study, where the rationale is to longitudinally follow up on previous interviews performed with HTRs at their one-year examination, in order to deepen the understanding of the meaning of surviving HTx. HTRs identified the transplantation as a transition point between illness and normality, although they recognized that they were in need of further care [13]. Our basic assumption was that surviving HTx was a transitional process involving moving from a profound sense of uncertainty [14] to something unknown, while dealing with self-efficacy [15] and the self-management expectations from transplant professionals, who often recommend support strategies that organ recipients do not always like or need [16].

What we already know about the inside perspective of HTRs is that being in uncertainty might be a source of great distress at one-year post-transplant, including doubting survival, doubting the recovery process, doubting one’s performance, struggling with close relationships, feeling abandoned and doubting the future [14]. Doubting recovery meant being unable to interpret symptoms, complications and side effects that in some cases made the recipient feel worse after the transplantation than before. Having a new heart means the necessity of relearning the signals from both the body and the heart. The HTRs stated that the new heart did not feel or react in the way they were used to [14]. Post-transplant complications, setbacks and side effects caused uncertainty, thereby negatively affecting the recipient’s performance, accomplishment and self-efficacy [15]. The HTRs had expected that they would recover faster than they did, leaving them pondering if they would ever fully recover. Pre-transplant MCS was a mediator for outcome expectations, as the initial post-transplant period was more difficult for some, compared with their recovery after MCS [15], leading to incongruence between experience and expectation. The core of self-efficacy [17] in the context of HTx [15] seemed to be the ability to balance expectations in order to avoid frequent disappointments, i.e., adjusting expectations to the present performance ability. As illustrated by Mishel’s framework [18] of uncertainty in illness, this meant reducing uncertainty in order to enhance self-efficacy. With this in mind, the aim was to explore the meaning of surviving three years after HTx compared to one year and to identify what constitutes the transition process.

## 2. Patients and Methods

A phenomenological-hermeneutic approach based on Ricoeur’s philosophy [19,20] was chosen to reveal the patients’ lived experiences, as well as to interpret and understand the meaning of surviving three years after a HTx. The phenomenological-hermeneutic method developed by Lindseth and Norberg [21] was used to perform the analysis.

This multi-center study was carried out at the two hospitals in Sweden where HTx is performed. Follow-up interviews were planned with the 14 HTRs who participated in the previous interview study at the time of their 12-month examination. Of those one had died, thus a total of 13 HTRs who had survived three years after HTx were invited to participate in this three-year follow-up study and 12 accepted, while one did not respond to the invitation. They had all previously participated in a study at the time of their 12-month follow-up where persons who were medically unstable, had limited knowledge of the Swedish language or were under the age of 18 years were excluded. The results of the first interviews have been published [14,15]. This is the longitudinal follow-up after another two years in order to grasp the meaning of the transition. The 12 persons who were interviewed had a mean age of 51.25 years (range: 30–66 years). They received both written and oral information on several occasions before providing their written informed consent. The characteristics of the informants are presented in Table 1.

### 2.1. Data Collection

The interviews were performed during 2017 and 2018, either at the transplant unit on the occasion of the 3-year follow-up or by telephone if living a long distance from the treating hospital and undergoing the follow-up at a hospital closer to home. All interviews, which consisted of reflective and open-ended questions, were digitally recorded and transcribed verbatim shortly afterwards [22]. The starting point of the interviews was a short recapitulation of the one-year interviews, after which the informants were asked to describe their experiences of surviving for a further two years by answering the following question: “What have been your main concerns during the last two years?” and follow-up questions such as “Can you please describe…?” or “Can you please explain about…?” to clarify and avoid misunderstanding. Questions related to interpretation from the previous study were also asked in order to deepen and widen the understanding. The interviews lasted for a mean time of 64 min (range: 34–98 min), resulting in 314 pages of transcribed text.

### 2.2. Data Analysis

The first step, naïve reading, consisted of reading each interview several times in order to grasp its meaning. The second step was structural analysis, which aimed to capture the meaning of lived experience by identifying and formulating themes [21]. In this step meaning units were identified, condensed, brought together and grouped into subthemes and themes (Table 2, Table 3).

The third and final step, comprehensive understanding, was performed by the authors reading the interview texts again and then reflecting together on the identified themes concerning the meaning of surviving for three years after a HTx and the change that had occurred since the first set of interviews two years before. Thus, the interpretation of the results was guided by the researchers’ pre-understanding from the one-year interviews, as well as their experience of caring for patients who have undergone HTx or advanced heart surgery.

### 2.3. Ethical Considerations

The study was approved by the Regional Ethics Board of Lund (Dnr. 2014/670-14/10) and conforms to the principles outlined in the Declaration of Helsinki [23].

## 3. Results

The *naïve* understanding revealed that the HTRs had strong feelings of acceptance of their life situation and that time had enabled this acceptance of limitations through adaptation. Therefore, the thematic structural analysis covers six themes illustrating the meaning of acceptance and adaptation, i.e., accepting life as it is, adapting to post-transplant limitations, adapting to a changed body, social adaptation, showing gratitude and feeling trust. Time was a key factor for being able to move on in life and test one’s bodily limits and performance ability. The awareness of life being limited meant that the future was not taken for granted and they adapted by living from day to day and avoiding long-term planning. It was also about adjusting everyday life and trying to return to normal life, thus achieving normalcy. They were grateful for the new heart and that their pre-transplant medical condition had been treatable, leading to a second chance in life. As time went by, they reached a state of acceptance that they would never be fully restored or that the body was not perceived as young anymore despite the new heart. Adaptation took place by choosing different priorities and eventually achieving acceptance and finding satisfaction in life.

### 3.1. Structural Analysis

#### 3.1.1. Accepting Life as It Is

The three years that had passed since the HTx had led to a sense of living on borrowed time and at the same time getting a second chance in life. Satisfaction with life was achieved by appreciating small things, focusing on aspects that created meaning and joy. A way of adapting to their current life situation was to compare it with their pre-transplant condition. Feeling much better now, they tried to live their lives as normally as possible and fully tested their limits.

… and I know then he (the son) said: But why did you push the car? You should have waited. Well I said, I wanted to test myself. Because I think you must dare.//Otherwise… yes, as I said there’s no point. Should you walk around dreading what could happen?… because I mean it can, of course, anything can happen, you can be run over or something like that … but you cannot walk around dreading…//… all the time thinking about what could happen.//Because as I feel that I am living on borrowed time, well if I get ten years or twenty or thirty years, it is still a bonus.

(3)

They doubted their future, knowing that their life expectancy was short due to the transplant, and as a result wanted to use time as best, they could. A way of adapting was to live from day to day and re-evaluate what is essential in life. They also adjusted their work situation in order to find a balance. Not taking life for granted anymore was a way of accepting things as they were.

I have been thinking about working for two more years, then I’m sixty-three years old, so I wasn’t going to work to sixty-five and that… but I probably would have thought the same even if this hadn’t happened. But it has become clearer now. I might have worked 100 percent if this had not happened. But… yes, I think… obviously I’m thinking about the future, but I don’t take it for granted anymore.

(11)

#### 3.1.2. Adapting to Post-Transplant Limitations

Having survived for three years involved adapting to setbacks, side effects and complications mainly related to their immunosuppressive medication. They adjusted their life habits to avoid infections, followed dietary recommendations and made travel arrangements to fit their life as an HTR. Adjusting to the immunosuppression also meant better mastering the balance between health and illness because they often became severely ill when infected.

So, I think that somehow my immune system becomes stronger as time goes by. I feel a difference between now and before…/… A year ago, I think that I was sicker and got colds more often, had tonsillitis all the time… I think my immune system is a little better now so… But I try to stay away from the kindergarten and such, I pick up the kids outdoors, so I don’t have to go inside.

(12)

Adapting to lifelong medication consisted of both the burden of being adherent to vital drugs and coping with the sometimes debilitating side effects. Transplant-related complications, such as post-transplant stroke, chronic pain, post-transplant tracheal stricture or eye conditions, caused suffering and affected everyday life.

… I have pressure and a little pain on my left side... you usually do not have that, but I guess it is because they opened me twice, the Heartmate first and then the transplant. So, it’s probably nerve damage that causes… It hasn’t got any better, no. I have this kind of electric TENS device. It’s a little bumpy to have it and the wiring hanging on me, but I used it when I started trying to work.//Since then, I have access to lighter nerve patches that are pasted on the skin and that relieve the pain. It is instead of taking pain killers.

(7)

#### 3.1.3. Adapting to a Changed Body

The patients experienced both physical and mental changes that required a great deal of adaption. Some described successful adaption as no longer feeling restricted by their previous illness. However, there were also HTRs with persistent physical limitations. Accepting their changed body was a time-consuming and stepwise process, as they had to cope with limitations such as becoming out of breath while walking quickly or being unable to exercise to the limit. The physical limitations could also mean that they were unable to resume full time work. Well-being was achieved by keeping the body in shape, which was a useful adaptation strategy. The HTRs felt that they existed in an altered body and sometimes found it difficult to interpret the bodily signals and reactions. Adapting could also mean not accepting the transplanted heart as one’s own but talking about it as something foreign.

… I can feel like this… in the past I had my heart… but this (my body) is after all me and the heart is not… I can… I have at some point probably said my heart but usually I say, “the heart”, as after all, it is someone else’s heart that is sitting in my body. So, I think I will never be able to feel that it is my heart… but it is still keeping me alive, so I am very grateful.

(3)

Surviving for three years had an emotional impact on their daily life that required adaptation to persistent difficulties with mental fatigue. The patients experienced continuing problems with decreased cognition and impaired short-term memory that also affected work. They felt a need for the transplant professionals to take a holistic view of them and emphasized the importance of time to recover mentally.

Just being at work all by yourself is not too bad, it functions well. But when you must concentrate on what others say it gets pretty hard. It is better now when there is a lot of noise and stuff… which was a pain before. I’ve probably learned to just ignore it... brain fatigue, typically you hear all the sounds. I do that now too, because I hear everything that happens around here...

(10)

#### 3.1.4. Social Adaptation

Being an HTR had a huge impact on social life that demanded re-construction and re-orientation. Surviving meant feeling alone, acknowledging the fact that people around them could not fully understand what they had gone through and they soon experienced that others were oblivious to the HTx. At the same time, they wanted to be independent, not bother others or be restricted by frequent transplant care visits. One social adaptation strategy was to prioritize friends, where those who were supportive were appreciated, while those who were not were let go. The HTRs were taken care of by their families but realized that it was demanding for their relatives to stand by, without being an actual part of the transplant process.

The HTRs turned to patient organizations and peer support for inspiration, knowledge and social stimuli. Hearing about others’ positive experiences created hope and confidence. Annual follow-up and biopsies reminded them of the pre-transplant disease and triggered negative feelings about their health situation. Experiencing sometimes unrealistic expectations of recovery from others also caused uncertainty about their performance and health status. The regular assessment by the authorities, e.g., the Insurance Agency, gave them the impression that they were not considered healthy despite the HTx.

… I have a new heart, am I healthy or unhealthy now? It leans more towards being unhealthy.//For example, when I was at the bank to invest money… I can’t remember exactly why but it was something about filling out a health declaration. Then you are not classified as healthy.

(7)

#### 3.1.5. Showing Gratitude

They felt gratitude for having a treatable illness and were grateful to the donor and the healthcare professionals for the opportunity to live on and considered each day a new gift. They were lucky to have been born in the right era to survive. Having a guilty conscience about surviving the transplantation while other patients did not motivated them to take good care of the new heart. Promoting donation was one form of repayment. Another was presenting the patient perspective at various professional meetings, which created a positive feeling of giving something in return.

I tried to pay back, I have paid for my heart… I already paid for it in 2014 when I got the bill./But then I tried… I repaid a little… Last autumn I lectured at... the nurse association for cardiac intensive care nurses, when they had a congress here in XXX. So, I presented the patient perspective. I buzzed for two hours and there were people from all over the country.

(5)

#### 3.1.6. Trusting Oneself and Others

Surviving three years meant gradually letting go of uncertainty and trusting the heart more and more. It involved trusting the body, not being anxious during training, becoming increasingly confident and not worrying about rejection. Accepting that life gives no guarantees they were confident that they were doing the best they could. Making notes could be helpful for becoming more aware of the recovery process. They also felt that trusting the new heart involved relying on the transplant professionals’ judgment of their health status. Thus, reliance on the transplant care was crucial.

… every day I am filled with wonder… that it (the heart) is working. But at the same time, I also take it a little for granted./Now that it has been working for three years there is a great probability that it will work for five more years too, and so on and so on. So, every day your sense of security increases because nothing happened after all.

(5)

### 3.2. Comprehensive Understanding

Our comprehensive understanding stems from the longitudinal change and the differences in experiences of being an HTR between the one-year and the three-year follow-up (Table 4).

HTRs start the transitional journey immediately after transplantation. Being an HTR means being an uncertain human being. First, they experience the uncertainty inherent in chronic illness [18] due to developing end-stage heart failure. Then they become an HTR either via MCS or not. Being an HTR involves adapting to the key existential question: How long will this heart last? Without receiving any valid or trustworthy answer as no answer exists. The threat of graft rejection is highlighted when going through the biopsy procedure, which serves as a constant reminder of living on borrowed time. Uncertainty and distress increase when there are no reliable cues to cling to, leading to the risk of reduced self-efficacy. After one year, the HTRs are in a state of complete uncertainty, unable to balance their unmet expectations and frequent disappointments against their physical accomplishments and mental well-being. Self-efficacy, in terms of confidence to perform a behavior necessary to reach a desired goal, is absent or reduced [17]. Confidence concerns efficacy expectations, which in this context means expectations of physical performance accomplishment, which is the most influential self-efficacy enhancing factor. Absence of performance accomplishment one year after HTx is a source of disappointment that potentially generates uncertainty, emotional arousal and stress. High expectations might also be a source of uncertainty when they remain unfulfilled. The gap between expectations and performance should be understood as uncertainty that needs to be identified and alleviated. It is obvious that it is a time-consuming process to reach acceptance and a solid sense of self-efficacy after HTx, as it involves the courage to face and accept the reality and adapt in all dimensions of life. Adaptation and transition are an existentially lonely process, dependent on social support from relatives and friends, while lacking support from the transplant professionals who have medical expectations of the outcome and lack a profound understanding of the HTRs’ inside perspective and meaning making.

After three years the HTRs had developed a useful approach for coping with unmet expectations and frequent disappointments, thus were no longer in uncertainty about what is possible to achieve. Developing probabilistic thinking [18] and accepting that there are many options and opportunities in life that one can focus on act as a mediator of self-efficacy, where confidence stems from a trust in the heart as well as in the transplant professionals’ ability to predict the uncertain future. When HTRs have achieved a sufficient performance accomplishment they seem to find it easier to master setbacks and complications without losing too much self-efficacy. The prerequisites for self-management are thus better after three years than after one year. As self-efficacy is the driving force in self-management it might not be possible to expect HTRs to master all the necessary self-management demands and behavioral changes inherent in being an HTR until three years after HTx.

## 4. Discussion

### 4.1. Reflection on the Findings

Being an HTR means having a chronic condition. The HTRs had reached a state of accepting their life situation three years after HTx. Mishel [18] suggests that it is the uncertainty that evolves early in the illness that contributes to the disruption and fluctuation seen among many patients with chronic illness. Uncertainty may be a state in which an HTR can experience a transition to a new perspective with a higher order and more complex orientation towards life. This constitutes the starting point from which HTRs can re-organize their self-structure and find a new perspective on life. To achieve and maintain order and coherence one strives for control and predictability and there is an implicit expectation in transplantation medicine that the cause of an illness can be determined with certainty and that the illness can be controlled. However, there is a need to develop probabilistic and conditional thinking in order to create a new orientation towards life, including abandoning the expectation of continual certainty and predictability. According to Mishel [18], by adopting such probabilistic thinking the HTRs might accept that there are many options and opportunities in life that they can choose to focus on. This creates a need to redefine what is important in life. They can also learn to appreciate and accept the fragility and impermanence of life, which in time leads to a more balanced and stable existence. The main theme of acceptance reflected this reasoning.

Adapting to post-transplant limitations and a changed body is an inherent challenge for HTRs, which is also shown by Lundmark et al. [24] who reported that lung recipients strive to adapt to a new normality by employing strategies such as comparing, accepting and adjusting. There are numerous self-management demands recommended by transplant professionals, e.g., being sufficiently active, adhering to medication and dietary guidelines, protecting oneself from infections and the sun, managing symptoms and mastering one’s role and emotions. Fellow organ recipients constitute a vicarious experience, showing that it is possible to perform and recover, but the self-management support from professionals is not always person-centered or perceived as relevant by patients [16]. Additionally, chronic pain is a well-known problem reported in several recent studies of organ recipients [25,26,27] and requires improved clinical attention. That the physical perception is conflictive and that recipients experience strange sensations in their body is described by Palmar-Santos et al. [8]. When they had their original heart, they could “feel” the organ itself, triggered by clinical issues associated with heart failure. In contrast, they feel nothing with the transplanted heart, leading to an impression of not having a heart. The new heart must be thought about because it is not felt [8].

After three years, the existential solitude inherent in being an HTR was still present. Pool et al. [11] argue that transplanted patients experience long-term complicated grief with respect to the donor and disenfranchised grief that may not be sanctioned. This grief might have been inadvertently disregarded or downplayed as it is obviously difficult to understand for those who have not experienced a HTx. However, social adaptation had taken place and the HTRs no longer struggled with close and more distant relationships. As shown in several papers, [28,29,30] it is possible to reconstruct one’s social function after transplantation. Showing gratitude might stem from the phenomenon reported by Mauthner et al. [7], where HTRs felt an interconnectedness with the donor, even when the transplanted heart was perceived as an intruder or stranger. They imagined the donor and questioned who they themselves were now. Trust in the transplanted heart as well as the professionals’ prognosis is possible when performance is accomplished, and self-efficacy increases. The transition is finalized by adaptation and acceptance that result in a state of self-efficacy that is vulnerable and volatile, easily affected by reduced physical accomplishment or mental fatigue, but sufficient to enable satisfaction with life and a sense of security.

### 4.2. Methodological Considerations

The prospective design and the chosen method enabled identification of the transitional process of acceptance and adaptation and further deepened the understanding of uncertainty after HTx reported previously [14,15]. Another strength is that the interviews after one and three years were performed by the same researcher, which made it possible to recapitulate and deepen the questions, but with the risk of bias from the previous understanding. However, the analysis was conducted by the first author who was not involved in the first interviews and thereby not biased due to this pre-understanding. The main limitation was that the sample only included Swedish speaking HTRs of Swedish origin and thus fails to reflect the ethnic diversity that is increasingly becoming a reality in Swedish healthcare.

### 4.3. Implications for Practice

The comprehensive understanding revealed consequences for health promotion and long-term follow-up after HTx. The findings show that health can be promoted by supporting and facilitating adaptation and that a key message is that it takes time. A vital part of the health promoting activities should be to assess barriers for adaptation at every long-term follow-up. Based on this understanding, it is time to re-evaluate the content of self-management support to strengthen health behaviors regarding dietary restrictions, medication adherence and sun protection, while taking the HTRs’ adaptation strategies and the transitional process into account. Self-management support must be adjusted to fit the HTRs’ physical, mental, social and existential transition. Thus, supporting transition through adaption should be the priority of self-management support after HTx in order to ensure that HTRs find a balance on the thin line between uncertainty and confidence in life, where self-efficacy serves both as a mediator and moderator. The findings in this study might be considered generic to a great extent. The understanding of how self-efficacy serves as both a mediator and a moderator along with the key role of uncertainty when developing self-efficacy, is useful in every encounter with persons suffering from illness or distress.

## 5. Conclusions

Achieving acceptance and a solid sense of self-efficacy after HTx is a time-consuming process that involves the courage to face and accept the reality and adapt in all dimensions of life. Promoting transition through adaption should be the priority of self-management support after HTx.

## Figures and Tables

**Table 1:** Patient characteristics (n = 12).

Characteristics	Number of Participants
Male	9
Female	3
LVAD	6
Dilated cardiomyopathy	9
Uni ventricular heart	1
ARVD/C (cardiomyopathy)	1
Ischemic cardiomyopathy	1

**Table 2:** Example of the structural analysis.

Meaning Unit	Condensation	Sub-Theme	Theme
“So… about a year ago, I got a whim. Then we sold our house and bought a farm down in XXX with animals and horses and everything possible there. So now I have regained my desire for life.”	Regaining zest for life	Enjoying life	Accepting life as it is
“No, I have done that, and I have got into such good shape, so I enjoy it. But the actual time that remains. I have already used up three years, I mean… purely statistically, it does not look good… after all. It doesn’t. But, I think it is sixty-five percent that can handle ten years, I mean, that… it’s a little over fifty percent, what the… I mean I have… seven years left. If I have seven years left, I do not want to go down there (to work) Monday, Tuesday and Wednesday and feel uncomfortable with those xxx years. That’s what I think.	Thinking about the quality of the time he is statistically expected to survive	Expecting a shorter life span	Accepting life as it is

**Table 3:** The structural analysis of the meaning of acceptance by adaptation.

Sub-Themes	Themes
Adjusting and enjoying life Living normally and fully Feeling recovered Expecting a shorter life span Doubting the future	Accepting life as it is
Suffering from complications Adjusting or not to the lifelong medication Adjusting to the risk of infection Mastering more severe illness when infected Balancing between health and illness	Adapting to post transplant limitations
Accepting a changed body and its limitations Keeping in good physical shape Not feeling restricted Experiencing mental fatigue and needing support Needing time to recover mentally	Adapting to a changed body
Feeling alone Wishing for independence Being supported Reducing one’s social network Needing a social context	Social adaptation
Feeling grateful Feeling responsible Paying back Feeling lucky	Showing gratitude
Trusting the new heart Feeling confident Trusting the transplant professionals	Trusting oneself and others

**Table 4:** The change in the meaning of being a heart recipient from one year to three years post-transplantation.

Main Theme One Year after Heart Transplantation: Being in Uncertainty	Main Theme Three Years after Heart Transplantation: Achieving Acceptance by Adaptation
Themes one year after heart transplantation (n = 14):	Themes three years after heart transplantation (n = 12)
Doubting survival	Accepting life as it is
Doubting the recovery process	Adapting to post transplant limitations
Doubting one’s performance	Adapting to a changed body
Struggling with close relationships	Social adaptation
Feeling abandoned	Showing gratitude
Doubting the future	Trusting oneself and others
